# The Leaf Extract of *Coccinia grandis* (L.) Voigt Accelerated In Vitro Wound Healing by Reducing Oxidative Stress Injury

**DOI:** 10.1155/2021/3963510

**Published:** 2021-01-06

**Authors:** Poommaree Namchaiw, Yamaratee Jaisin, Cholticha Niwaspragrit, Kittiya Malaniyom, Anyamanee Auvuchanon, Piyanee Ratanachamnong

**Affiliations:** ^1^Biological Engineering Program, Faculty of Engineering, King Mongkut's University of Technology Thonburi, Bangkok, Thailand; ^2^Neuroscience Center for Research and Innovation, Learning Institute, King Mongkut's University of Technology Thonburi, Bangkok, Thailand; ^3^Department of Pharmacology, Faculty of Medicine, Srinakharinwirot University, Bangkok, Thailand; ^4^Expert Center of Innovative Agriculture, Thailand Institute of Scientific and Technological Research (TISTR), Pathum Thani, Thailand; ^5^Department of Horticulture, Faculty of Agriculture at Kamphaeng Saen, Kasetsart University, Nakhon Pathom, Thailand; ^6^Department of Pharmacology, Faculty of Science, Mahidol University, Bangkok, Thailand

## Abstract

The impairment in the regulation of the physiological process in the inflammatory phase of wound healing results in oxidative stress damage, which increases the severity and extends the healing time. In this study, we aimed to evaluate the radical scavenging properties of *Coccinia* leaf extract and its ability to ameliorate a migration process in vitro. *Coccinia* is a medicinal plant that was used in ancient times for relieving insect bite itching and swelling. However, the role of *Coccinia* leaf extract as an antioxidant related to the process of wound healing has never been studied. In this study, we demonstrated that the leaf extract possessed antioxidant properties that acted as a proton donor to neutralize reactive oxygen species with the IC_50_ value of 4.85 mg/mL of the extract. It could chelate iron with the IC_50_ value of 21.39 mg/mL of the extract. The leaf extract protected the human fibroblasts and keratinocytes from hydrogen peroxide-induced oxidative stress by increasing cell survival rate by more than 20% in all test doses. The protective property was dose-dependently correlated with the decrease in reactive oxygen species formation. In addition, the leaf extract enhanced the cell migration rate of fibroblasts and keratinocytes up to 23% compared with vehicle control. The results suggested that *Coccinia* leaf extract may be a potential herb for increasing the wound healing process with its antioxidant capacity and can be used as an herbal ingredient for the utilization of skincare products.

## 1. Introduction

The skin is the largest organ in the body. It is the first barrier to protect the internal organs from external penetration. The injury that breaks the skin that is so-called “wound” can be an acute or chronic injury, depending on the time course of the wound healing process. The physiological pathway of wound healing is characterized by the cascade of associated events including hemostasis, inflammation, proliferation, and remodeling [1]. These phases are overlapping, continuous, and indistinguishable. Following the hemostatic phase to stop the outflow bleeding, platelets, the key players, release several chemotactic factors to induce the migration of leukocytes to the site of injury [1, 2]. The inflammation phase continues as leukocytes arrived at the lesion, encountered the invading pathogen, and scavenged cellular debris [2]. During this phase, the inflammatory cells secrete a number of cytokines and growth factors that lead to fibroblast and keratinocyte migration and initiate the next stages of wound healing: proliferation phase and remodeling phase [1–3]. As the inflammatory process continues, multiple cells are attracted to the site of injury and initiate the release of reactive oxygen species (ROS) and protease to get rid of the pathogens. Oxidants such as superoxide anions, hydroxyl radicals, and hydrogen peroxides are secreted by leukocytes, endothelial cells, and vascular cells [4]. The low concentration of oxidants acts as secondary messengers that modulate several downstream signaling pathways including inflammation and wound repairing process [4, 5]. Although the secretion of oxidants has a protective role, the dysregulation of an inflammatory response such as overproduction of oxidants is cytotoxic and may contribute to a chronic wound [4]. The prolonged inflammation phase results in a chronic wound, in which the patient encounters pain and may develop an infected wound.

Keratinocytes and fibroblasts are the principal cells that reside in the epidermis and dermis, respectively [6]. Keratinocytes provide the barrier that prevents water loss and protects the dermis from penetration, while fibroblasts produce an extracellular matrix to provide the structural supportive network for surrounding cells [3]. Upon skin injury, both of them are recruited to the lesion site then undergo the morphological and functional changes to drive the granulation tissue formation, which ultimately regenerates the damaged tissue [6, 7]. Previous studies revealed that the function of keratinocytes and fibroblasts can be impaired by excessive oxidative stress during the prolonged inflammation phase, which results in an unhealed wound [8, 9]. The cost of wound management is rising as it progresses toward being a chronic wound. Thus, several attempts are aimed at limiting the inflammation before it develops toward a chronic, nonhealing wound.

The *Coccinia grandis* (L.) Voigt, commonly known as *ivy gourd*, is a vegetable grown in subtropical and tropical areas of South East Asia, South Asia, and Africa [10]. It is a climbing and ground-creeping plant that spreads rapidly, requires low maintenance, and is highly naturalized. Its fruits and leaves are edible either fresh or cooked and are used in cooking and as a household remedy from time to time [11–14]. In South East Asia, *ivy gourd* leaves are used as antibruises and anti-itching from insect bites by applying the lesion with crushed fresh leaves [15]. Previous studies showed that *ivy gourd* leaf extract is a good source of proteins, minerals, vitamins, and other phytochemicals (e.g., polyphenol, flavonoid, saponin, and sterol contents) [15, 16]. Thus, it was added to meals for their medicinal effects such as antioxidant, antiulcer, anti-inflammatory, antidiabetic, and analgesic potential [13, 14, 17–20]. In addition, the potential effect on wound healing was recently observed in animal models [21, 22]. However, the mechanisms of action related to wound healing have not been determined. In this study, we demonstrated the antioxidant properties of *Coccinia* leaf extract which reduced oxidative stress injury in human fibroblast and keratinocytes and its potential effect on human fibroblast and keratinocyte cell migration.

## 2. Materials and Methods

### 2.1. Chemicals and Equipment

Methanol (CH_3_OH) was purchased from RCI Labscan, Thailand. Gallic acid monohydrate (C_7_H_6_O_5_·H_2_O) and sodium carbonate anhydrous (Na_2_CO_3_) were purchased from Riedel-de Haën, Germany. Folin-Ciocalteu's phenol reagent and ethanol (C_2_H_5_OH) were purchased from Merck, Germany. Thiazolyl Blue Tetrazolium Bromide (MTT), allantoin, 2′,7′-dichlorofluorescin diacetate, quercetin (C_15_H_10_O_7_), aluminum chloride (AlCl_3_), potassium acetate (CH_3_CO_2_K), ferrous sulfate heptahydrate (FeSO_4_·7H_2_O), and 3-(2-pyridyl)-3,0-diphenyl-1,2,4-triazine-4-4′-disulfonic acid sodium salt (C_20_H_13_N_4_NaO_6_S_2_) were purchased from Sigma Chemical Company, USA. Dulbecco's modified Eagle medium (DMEM), heat-inactivated FBS, Non-Essential Amino Acids Solution (100x), and 0.25% trypsin-EDTA were purchased from Gibco, USA. Hydrogen peroxide and non-essential amino acid were purchased from Merck, USA. The scratch wound healing images were taken under an inverted microscope (Olympus, Japan), and the DCFH-DA assay was taken under a confocal fluorescent microscope (Olympus, Japan). The absorbance and fluorescence were evaluated with a microplate reader (BioTek Instruments, USA).

### 2.2. Plant Material and Extraction

*Ivy gourd* leaves were collected from Pathum Thani agricultural plot, Thailand. The voucher specimen has been deposited at the Herbarium of Forest Botany Division, Department of National Parks, Wildlife and Plant Conservation, Thailand. The dried leaves (50 g) were extracted with 95% methanol at room temperature. The solvent was renewed every 48 hours three times. The extract was filtered and concentrated under the reduced pressure rotary evaporator. The dried sample was kept in desiccators until performing further experiments. The methanol leaf extract sample used in cell line treatment was diluted with DMSO (final concentration of 200, 100, and 50 *μ*g/mL in 0.1% DMSO). Vehicle control was 0.1% DMSO.

### 2.3. Chemical Investigation

#### 2.3.1. Total Phenolic Contents

The total phenolic content of the leaf extract was determined by the colorimetric method as described by Slinkard and Singleton [23]. In brief, 30 *μ*L of methanol leaf extract or the reference standard gallic acid was dissolved in 790 *μ*L deionized water. The mixture was then mixed with 50 *μ*L Folin-Ciocalteu's phenol reagent and incubated at room temperature for 5 minutes before adding 150 *μ*L saturated Na_2_CO_3_ and further incubated at room temperature for 50 minutes. The total polyphenol contents were measured at a wavelength of 750 nm and expressed as milligram gallic acid equivalents (GAE) per gram of extract.

#### 2.3.2. Total Flavonoid Contents

The total flavonoid content was determined by the aluminum chloride colorimetric method as described by Chang et al. [24]. In brief, 100 *μ*L extract or reference standard quercetin solution was dissolved in 560 *μ*L deionized water, 20 *μ*L of 1 M potassium acetate, and 300 *μ*L 95% ethanol. The mixture was mixed with 100 *μ*L of 10% aluminum chloride then incubated at room temperature for 30 minutes. The total flavonoid content of the leaf extract was measured at a wavelength of 415 nm. The amount of total flavonoid of the methanol leaf extract was expressed as quercetin equivalents (QE).

### 2.4. Evaluation of Antioxidant Activity

#### 2.4.1. DPPH Free Radical Scavenging Assay

The free radical scavenging activity was measured by DPPH scavenging assay. DPPH is a stable free radical and a strong purple color which can be measured by a spectrophotometer [25]. In the presence of antioxidant compounds, the DPPH became discolored by receiving an electron or hydrogen atom. To identify the antioxidant property of the leaf extract, the decrease of DPPH absorbance was determined. Briefly, 20 *μ*L of the extract was mixed with 180 *μ*L DPPH solution in a 96-well plate and the absorbance was measured at 515 nm. Trolox, a vitamin E derivative, was used as a reference. The antioxidant activity was calculated as the percentage of scavenging activity.

#### 2.4.2. Ferrous Ion Chelating Activity

50 *μ*L of 5 mM ferrozine was mixed with 25 *μ*L of 2 mM FeSO_4_. Ferrozine is a chromophoric complexing agent that selectively binds with ferrous ions to form a stable water-soluble compound, which has an absorbance at 562 nm. To determine the chelating activity, the leaf extract was added into the reaction and the decrease of absorbance was evaluated. EDTA, a versatile chelating agent, was used as a reference.

### 2.5. Cell Culture

Human fibroblast cell line HFb (ATCC® CRL-2429™, USA) was purchased from ATCC, USA. Human keratinocyte cell line HaCaT was purchased from Cell Lines Service, Germany. HFb was grown in DMEM with high-glucose complete medium and supplemented with 10% heat-inactivated FBS. HaCaT was grown in DMEM with high-glucose complete medium and supplemented with 10% heat-inactivated FBS and 5 mL of Non-Essential Amino Acids Solution. The cultures were rinsed with PBS solution before dissociated with 0.125% trypsin-EDTA. The cell suspensions were plated and cultured in a plastic 75 cm^2^ flask at 37°C, 5% CO_2_. After seeding, the cells were allowed for growth for 24 hours before performing further experiments.

### 2.6. Cell Viability

To investigate the nontoxic concentrations of the methanol extract and a toxic concentration of H_2_O_2_, cells were seeded on 96-well culture plates with a density of 25 × 10^3^ cells/well and incubated at 37°C, 5% CO_2_ overnight. The various concentrations of the extract and hydrogen peroxide were treated on cell culture for 24 hours, and cell viability was determined using the MTT assay; the absorbance was measured at 540 nm by a microplate reader. The protective effect of the leaf extract on hydrogen peroxide-induced cytotoxicity was performed by treated cell culture with the extract for 2 hours before treatment with hydrogen peroxide. The percentage of cell viability was calculated relative to vehicle control.

### 2.7. Evaluation of Intracellular Reactive Oxygen Species by DCFH-DA Assay

The experiment was performed according to the manufacturer's recommendation (Invitrogen, CA, USA). In brief, this assay is based on the oxidation of the nonfluorescent probe 2′,7′-dichlorofluorescin diacetate (DCFH-DA) by intracellular ROS to yield the highly fluorescent 2′,7′-dichlorofluorescein (DCF). Keratinocytes (HaCaT) and fibroblasts (HFb) cells were seeded on a 96-well plate at a density of 25 × 10^3^ cells/well in 10% FBS in DMEM medium and cultured at 37°C, 5% CO_2_ overnight. After that, cells were treated with the leaf extract for 2 hours before hydrogen peroxide was added and further incubated at 37°C, 5% CO_2_ overnight. The medium was then removed and stained with DCFH-DA for 1 hour. The staining medium was then removed and followed by one time washing with 1x PBS buffer, and the fluorescence intensity was immediately analyzed at excitation and emission of 485 and 528 nm. The fluorescence intensity reflected the intracellular ROS formation. The data were represented as fold changes relative to vehicle control, and the generation of ROS was analyzed in each cell separately.

### 2.8. Evaluation of the Effect of *Coccinia* Leaf Extract on Scratch Wound Healing

Human keratinocyte and fibroblast were seeded on a 24-well plate with a density of 2 × 10^5^ cells/well and cultured at 37°C, 5% CO_2_ overnight. The adherent cell layer was scratched with a sterile tip and treated with various concentrations of the leaf extract. The images of the scratched area were captured at 15 hours after scratching and then analyzed with the ImageJ software (National Institutes of Health, US). The percentage of cell migration to cover the scratched area was calculated and presented as a relative number to time zero.

### 2.9. Statistical Analysis

The chemical analysis of total phenolic and flavonoid contents was measured 10 replicates of multiple concentrations. The average was plotted to make a standard curve and the number of chemical equivalents to GAE and QE was calculated, respectively. Other experiments, one-way ANOVA with Dunnett's post hoc analysis of 6 replicates, were used to perform the statistical analysis.

## 3. Results

### 3.1. Polyphenolic and Flavonoid Contents

Polyphenolic and flavonoid compounds are the chemical structure containing multiple hydroxyl substituents on an aromatic ring. Due to their structure, polyphenol compounds are good electron and proton donors. They are capable to scavenge free radicals and reduce oxidative stress by transferring H-atom from their hydroxyl group(s) to free radicals [26]. Previous studies revealed that the possession of multiple hydroxyl groups that attached to the aromatic ring manipulated the antioxidant activity as a free radical scavenging and metal ion chelating site; the more they have hydroxyl groups the more they are antioxidant [26, 27]. In this study, we showed that *Coccinia* leaf extract contained a polyphenolic content of 104.88 ± 0.8 mg GAE equivalent per gram of extract and flavonoid content of 35.35 ± 1.82 mg QE equivalent per gram of extract. Therefore, the presence of polyphenolic and flavonoid contents in the leaf extract may contribute to its antioxidant activity.

### 3.2. Antioxidant and Ferrous Chelating Activities

**Figure 1:**
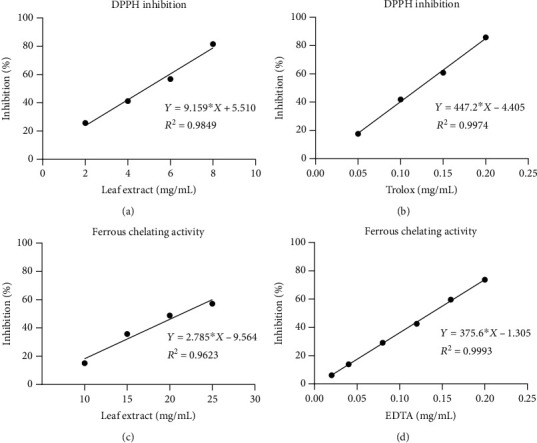
The determination of the antioxidant activity of (a) the leaf extract and (b) Trolox. Graphs plotted for the concentration of substance in mg/mL versus percentage of the inhibition of DPPH. The determination of ferrous chelating activity of (c) the leaf extract and (d) EDTA. Graphs plotted for the concentration of substance in mg/mL versus percentage of ferrous ion chelating effect.

The antioxidant property of *Coccinia* leaf extract was determined by DPPH scavenging assay compared to a standard, vitamin E derivative (Trolox). The leaf extract exhibited DPPH radical scavenging effect in a dose-dependent manner. The IC_50_ value of the leaf extract was 4.85 mg/mL or equaled to 98.96 *μ*mol Trolox equivalents per gram of extract (Figures 1(a) and 1(b)). The ability of DPPH radical scavenging indicated its ability to donate protons to DPPH and subsequently neutralize free radicals. Along with the generation of ROS during inflammation, iron is part of an interplay involving the generation of hydroxyl radicals and superoxide anions from iron redox cycling reaction (Fenton and Haber-Weiss reaction) [28, 29]. We showed that the leaf extract possessed an iron chelating property. Relative to the chelating agent, EDTA, *Coccinia* leaf extract had a ferrous ion chelating property with an IC_50_ value of 21.39 mg/mL or equaled to 22.39 *μ*mol EDTA equivalent per gram of extract (Figures 1(c) and 1(d)), suggesting that the leaf extract may lower the metal-induced radical formation and subsequently prevent the cellular oxidative damage in the prolonged inflammation.

### 3.3. Protective Effect of *Coccinia* Leaf Extract on Hydrogen Peroxide-Induced Oxidative Injury

**Figure 2:**
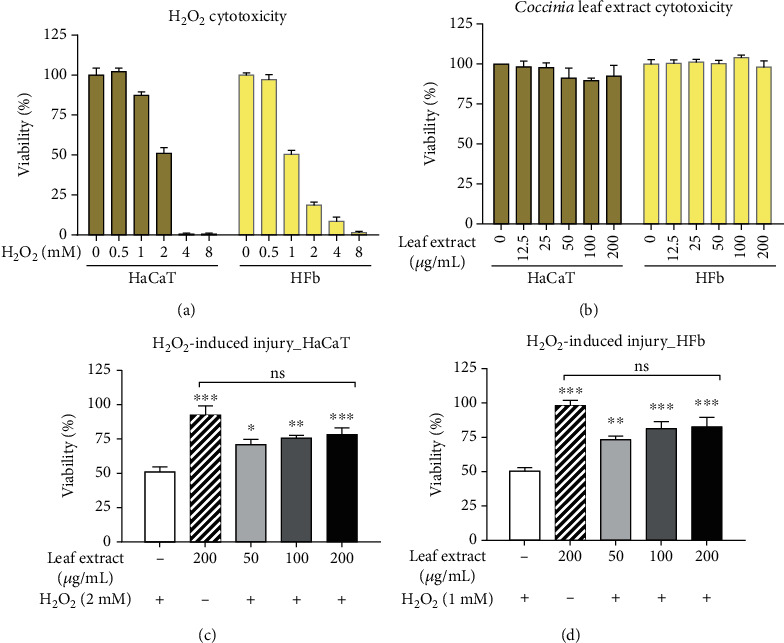
The graphs showed the viability of human keratinocyte, HaCaT, and human fibroblast, HFb, cell lines upon (a) hydrogen peroxide (H_2_O_2_) treatment and (b) the leaf extract treatment. The capability of the leaf extracts to rescue the hydrogen peroxide-induced oxidative stress and cytotoxicity was employed in (c) HaCaT cell line and (d) HFb cell line. The data were analyzed with one-way ANOVA with Dunnett post hoc analysis, *n* = 6. ^∗^*P* < 0.05, ^∗∗^*P* < 0.01, and ^∗∗∗^*P* < 0.001 compared to hydrogen peroxide-treated condition (+H_2_O_2_). Besides, the treatment of 200 *μ*g/mL methanol leaf extract following hydrogen peroxide-induced oxidative stress and cytotoxicity was not significantly different (shown as “ns”) compared with no hydrogen peroxide induction (-H_2_O_2_).

**Figure 3:**
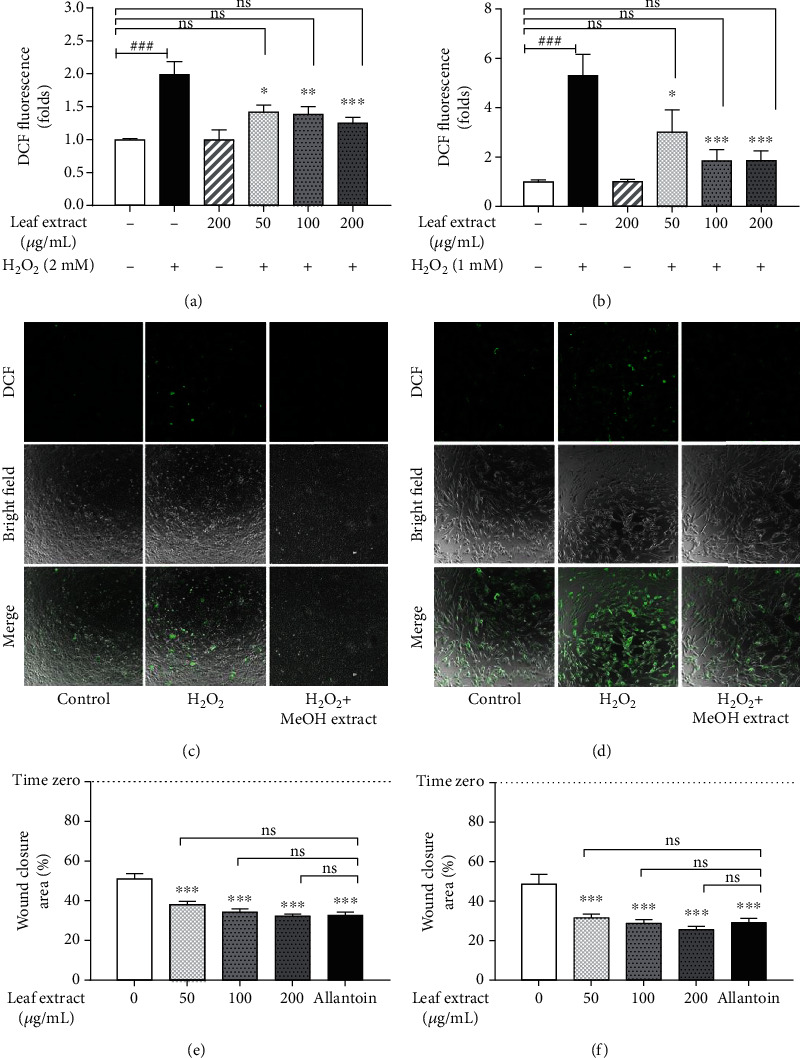
The effect of *Coccinia* leaf extract on the reduction of reactive oxygen species upon hydrogen peroxide-induced oxidative stress and cytotoxicity was employed by DCF fluorescence staining in (a, c) HaCaT cell line and (b, d) HFb cell line. The fluorescence intensities were expressed in fold changes compared to vehicle control (-H_2_O_2_-leaf extract). The effect of leaf extract on the migration of (e) HaCaT cells and (f) HFb cells was shown as a wound closure area or the remaining area uncovered by the cells. The scratch-wound closure was monitored fifteen hours later. The scratch area at time zero was set to 100%. The data were analyzed with one-way ANOVA with Dunnett post hoc analysis, *n* = 6. The methanol leaf extract treatments (+H_2_O_2_+leaf extract) showed as ^∗^*P* < 0.05, ^∗∗^*P* < 0.01, and ^∗∗∗^*P* < 0.001 compared with hydrogen peroxide treatment (+H_2_O_2_). Besides, the methanol leaf extract treatment at all range of concentrations (+H_2_O_2_+leaf extract) showed no significant difference (“ns”) from vehicle control (-H_2_O_2_-leaf extract).

In this study, hydrogen peroxide, H_2_O_2_, was used to induce cell death. Human fibroblasts and keratinocytes were used to determine the sublethal dose of H_2_O_2_ (Figure 2(a)). We found that human fibroblast was more sensitive to H_2_O_2_-induced cytotoxicity than human keratinocytes. The treatment of 1 mM H_2_O_2_ on fibroblast cells led to 50.5 ± 2.45% viability, while keratinocyte exhibited 51.14 ± 3.52% viability upon 2 mM H_2_O_2_ treatment. The treatment of *Coccinia* leaf extract showed negligible cytotoxicity and minimal cell proliferative capacity on both cell lines all over the range of the extract concentrations (Figure 2(b)). The selected sublethal hydrogen peroxide dose was used to treat each cell line, and the capability of the leaf extract to rescue the hydrogen peroxide-induced cytotoxicity was monitored. H_2_O_2_ treatment alone (negative control) was used to induce oxidative stress damage in the cell, while *Coccinia* leaf extract treatment alone (positive control) showed minimal cytotoxicity in the cell (Figures 2(c) and 2(d)). Upon the treatment of the extract, the cytotoxicity of H_2_O_2_-induced oxidative damage was recovered in a dose-dependent manner in both cell lines. The treatment of 200 *μ*g/mL of the extract could rescue the cytotoxicity more than 27% compared with negative control, and the recovery was found to have no significant difference from the positive control. Together, this suggested that *Coccinia* leaf extract could ameliorate the human fibroblast and keratinocyte oxidative stress induced by H_2_O_2_. Next, we further tested whether the protective effect of the leaf extract relates to the intracellular ROS scavenging or else. We stained the cells with a cell-permeant dye, DCFH-DA, and determined the fluorescence intensity (Figures 3(a) and 3(b)). The amount of intracellular ROS was showed in relation with vehicle control treatment, 0.1% DMSO. Oxidative stress injury in human fibroblasts was induced by 1 mM H_2_O_2_, while oxidative stress injury in human keratinocytes was induced with 2 mM H_2_O_2_. The relative amount of fluorescence was analyzed in each cell line separately. Compared with vehicle control, the treatment of *Coccinia* leaf extract did not increase the intracellular ROS production in both cell lines, while H_2_O_2_ could induce ROS generation about two times more in human keratinocytes and five times more in human fibroblasts. This also correlated with the previous results showing that human fibroblast was more sensitive to oxidative stress injury than keratinocytes, even when they were treated with the lower H_2_O_2_ dose. We found that the treatment of the leaf extract reduced cellular ROS in both cell lines. The decrease of cellular ROS was observed even in the lowest test dose (50 *μ*g/mL), and it continued to decrease ROS in a dose-related manner. Upon the supplement of the highest test dose (200 *μ*g/mL) of the extract onto H_2_O_2_-induced ROS generation, the amount of cellular ROS was not significantly different from no oxidative stress damage (the condition of no H_2_O_2_ treatment), suggesting that the leaf extract could scavenge the intracellular ROS in both cell lines, in which its protective effect might correspond with the cellular oxidative stress (Figures 3(a)–3(d)).

### 3.4. *Coccinia* Leaf Extract Induced Cell Migration Revealed by Scratch Wound Healing Assay

In the attempt to evaluate the wound healing capacity, the scratching on fibroblasts and keratinocytes was employed to determine the effect of *Coccinia* leaf extract. The migration property of the extract was compared with a commercialized wound care drug: allantoin. The coverage area of the scratch was calculated and expressed as the percentage relative to time zero (accounted as 100% scratching area). The scratching area of untreated human fibroblast and human keratinocyte monolayer is shown in Figures 3(e) and 3(f), respectively. We demonstrated that the leaf extract induced human fibroblast and keratinocyte cell migration in a dose-related capability. The lowest test dose, 50 *μ*g/ml extract, was observed to reduce the scratched area up to 13% in keratinocytes and 17% in fibroblasts compared to vehicle control treatment (0.1% DMSO), whereas the maximum dose of 200 *μ*g/mL leaf extract enhanced the cell migration, which reduced the scratched area by about 19% in keratinocytes and 23% in fibroblasts. We found that the potential effect of the leaf extract on wound healing assay was similar to allantoin at all ranges of test doses. This effect was observed in both cell lines. Together, this indicated that the *Coccinia* leaf extract efficiently enhanced the migratory capacity of cells, which accelerated the wound healing process.

## 4. Discussion

Upon tissue damage, several cascades respond to the injury and alleviate the suffering. One of those cascades is the inflammatory process, which is aimed at eliminating the invading pathogen, getting rid of tissue debris, and secreting secondary messengers to initiate the downstream pathways. In the normal wound healing, the ROS such as superoxide radicals, hydroxyl radical, and hydrogen peroxide act as the secondary messengers to regulate downstream pathways such as tissue inflammation and proliferation [30]. The generations of reactive oxygen species and reactive nitrogen species are normal defense mechanisms following the inflammatory process. Due to the short half-life of superoxide anions, they are catalyzed by superoxide dismutase to hydrogen peroxide, a relatively stable compound. Although hydrogen peroxide is safer than other oxidants, its accumulation during the prolonged stimulation of the inflammatory process can further regenerate hydroxyl radicals via the Fenton reaction. Iron is the abundant transition metal in our body. It is involved in the redox cycling pathway in the cell to generate hydroxyl radicals from hydrogen peroxide. A previous study showed that the imbalance of iron homeostasis has been described in several chronic inflammatory diseases [29]. Under the pathological process, the dysregulated, incomplete, or overactive inflammation results in abnormal wound healing such as scars, keloids, and chronic wounds. The balance between proinflammation and anti-inflammation needs to be well regulated to control the damage and minimize tissue injuries. Otherwise, the prolonged inflammation may result in an excessive free radical which further damages tissue. Therefore, the constriction of the inflammation stage was targeted to manage the severity of the injury [31–33].

Severe nonhealing wounds could lead to infection and amputation. The estimated prevalence of global chronic wounds was 2.21 cases per 1,000 population [34]. The cost of wound management was estimated to be around 28 to 96 billion US dollars annually [35]. The treatment of a wound appeared to be expensive, especially in chronic, nonhealing wounds which sustain the cost for a longer period. Due to the high economic burden, several countries increase the use of phytomedicine in chronic wounds as they have an affordable price. Although natural products possibly have higher safety concerns than chemical-based therapy, potential property, activity, and validation by scientific methods need to be determined. Previously, natural products such as honey, chamomile, rosemary, and turmeric were used as therapeutic agents for reducing inflammation in wound management [31, 36–38]. For instance, chamomile has been used as a wound care product under the trade name “*Kamillosan*.” It accelerates tissue epithelization which results in wound drying and also acts as an antioxidant and anti-inflammatory agent [39, 40]. The other well-known natural product for wound care is honey. Its medicinal properties are related to the effects of antioxidants and anti-inflammation that restricted the oxidative injury and advanced the wound healing process [41, 42]. The antioxidant properties of natural products from plants were related to the enrichment of polyphenol constitutes [43–48]. Due to the chemical structure of phenolic compounds, it scavenges free radicals by transferring electrons and H-atoms from their OH-group(s) to the free radicals thereby exhibiting antioxidant property [49]. Besides scavenging free radicals, phenolic compounds also scavenge nonradical reactive species such as hydrogen peroxide by donating electrons and H-atoms to hydrogen peroxide which can subsequently be converted to water molecules [27]. As a good electron and proton donor, the phenolic compound acts as a chelator of several transition metal ions such as zinc (Zn^2+^), iron (Fe^2+^), and copper (Cu^2+^) which is beneficial for lowering metal-induced oxidative stress injury. Moreover, previous studies found that the polyphenolic compound reduced the activity of enzymes involved in ROS formation [50–52]. Polyphenols also temper the immune response by modulation of inflammatory cytokines [53]. Besides the anti-inflammation and antioxidant properties of phenolic compounds, they have been found to enhance fibroblast migration which promotes the wound repairing process, although the mechanism of action has not been described yet [54].

Nowadays, the usage of plant-based medication is increasing. In this study, we were interested in the effect of *Coccinia* leaf extract on the modulation of oxidative stress damage and its related wound healing properties. In ancient times, this medicinal plant was used to reduce the swelling and itching from an insect bite. This suggested its ability to decrease the inflammatory process. Recent studies have revealed the metabolic profile of the *Coccinia* leaf extract and its ability to enhance wound healing in vivo [21, 22]. However, it has not been tested in the cell culture model yet, especially its antioxidant properties and its role on cell migratory effect. In this present study, we showed that the leaf extract possessed considerable polyphenolic and flavonoid contents. The leaf extract could rescue cell survival in hydrogen peroxide-induced oxidative stress damage. Following the tests of antioxidant assays, the leaf extract was capable to decrease free radicals and chelate the ferrous ions. Fibroblasts were very sensitive to the hydrogen peroxide-induced oxidative damage than keratinocytes. The cytotoxicity of fibroblast may result in the lack of extracellular matrix deposition, hampering wound contraction, and delaying tissue remodeling process. Here, we have first demonstrated that *Coccinia* leaf extract could reduce the oxidative stress damage in both human fibroblast and keratinocyte cell lines.

During the wound healing process, fibroblast and keratinocytes are attracted to the site of injury following the chemotactic secretion from inflammatory cells and are responsible for granulation and reepithelization of tissue. They initiate cell proliferation and tissue formation that occur in the late phase of inflammation till the remodeling phase of wound healing [1, 55]. Keratinocytes have released numbers of proteins to reconstitute the damaged epidermis and basement membrane, while fibroblasts undergo proliferation and modify their interaction with the extracellular matrix to recreate healthy granulation tissue [1, 55]. In this study, the wound healing property of the leaf extract was determined by scratch wound healing assay [56]. This reliable method has been described by Lampugnani in 1999 and was used in several previous studies in comparison to in vivo study models [57–60]. Upon the scratch wound assay, we showed that the *Coccinia* leaf extract could reduce the scratched area in both keratinocytes and fibroblast cells. This appearance could be either by the induction of cell migration to cover the scratched area or by the increase of cell proliferation, but we found that the *Coccinia* leaf extract did not increase the cell number at all test concentrations. Thus, we believed that the wound healing property of the *Coccinia* leaf extract was related to the modulation of cell migration. Interestingly, the leaf extract could induce the cell migration to a similar level as commercializing wound care product allantoin. The mechanisms of action of allantoin to promote wound healing were related to the modulation of an inflammatory response and the induction of fibroblast proliferation and reepithelization [61–63]. Our finding demonstrated that the potential effect of the *Coccinia* leaf extract on wound healing was related to its antioxidant properties and the induction of cell migration.

## 5. Conclusion

The accumulation of hydrogen peroxide is present in chronic wound healing as well as several chronic inflammatory diseases such as atherosclerosis, diabetes, and systemic inflammatory response syndrome. In this study, we showed that the *Coccinia* leaf extract could alleviate the cytotoxicity from hydrogen peroxide exposure, in vitro. It also had beneficial effects on wound healing process by promoting the migration of fibroblasts and keratinocytes. Due to the easiness and low cost of *Coccinia* maintenance, this could accommodate the usage of this medicinal plant as a natural antioxidant, anti-inflammatory agent, and wound care product. However, further study is needed to investigate the other wound healing mechanism of the leaf extract.

## Data Availability

All datasets generated during the study are included in the article.
